# Predictors of functional improvement and pain reduction in rheumatoid arthritis patients who achieved low disease activity with disease-modifying antirheumatic drugs: a retrospective study of the FIRST Registry

**DOI:** 10.1186/s13075-024-03369-8

**Published:** 2024-07-26

**Authors:** Sae Ochi, Koshiro Sonomoto, Shingo Nakayamada, Yoshiya Tanaka

**Affiliations:** 1 Department of Laboratory Medicine, The Jikei University School of Medicine, Nishishinbashi 3-25-8, Minato-ku, Tokyo 105-8461 Japan; 2 The First Department of Internal Medicine, School of Medicine, University of Occupational and Environmental Health, Japan, Iseigaoka1-1, Yahatanishi-ku, Kitakyushu, Fukuoka 807-8555 Japan; 3 Department of Clinical Nursing, School of Health Sciences, University of Occupational and Environmental Health, Japan, Iseigaoka1-1, Yahatanishi-ku, Kitakyushu, Fukuoka 807-8555 Japan

**Keywords:** Rheumatoid arthritis, Disease-modifying antirheumatic drugs, Health assessment questionnaire-disability index, Pain visual analogue score

## Abstract

**Background:**

Rheumatoid arthritis (RA) patients sometimes exhibit different levels of improvement in health assessment questionnaire-disability index (HAQ-DI) and subjective pain visual analogue score (VAS) even after achieving low disease activities (LDA). This study aimed to identify factors associated with improvement in HAQ-DI and pain VAS among those who achieved LDA.

**Methods:**

Data of the FIRST registry, a multi-institutional cohort of RA patients treated with biological and targeted-synthetic DMARDs (b/tsDMARDs) were analyzed. Patients who were enrolled from August 2013 to February 2023 and who achieved clinical LDA [clinical disease activity index (CDAI) ≤ 10.0] at 6 months after starting treatment were included. Multiple logistic regression analyses were conducted to identify the factors that associated with achieving HAQ-DI normalization (< 0.5), HAQ-DI improvement (by > 0.22), or pain VAS reduction (≤ 40 mm).

**Results:**

Among 1424 patients who achieved LDA at 6 months, 732 patients achieved HAQ-DI normalization and 454 achieved pain VAS reduction. The seropositivity and the use of JAK inhibitor compared with TNF inhibitor were associated with both HAQ-DI < 0.5 and pain VAS reduction at 6 months. On the other hand, older age, past failure in ≥ 2 classes of b/tsDMARDs, higher HAQ-DI at baseline, and use of glucocorticoid were associated with the lower likelihood of HAQ-DI normalization and pain VAS reduction. Longer disease duration, being female, and higher disease activity at baseline was negatively associated HAQ-DI normalization alone. Comorbidities were not associated with the outcomes.

**Conclusions:**

These results suggest some preferable treatment may exist for improvement of HAQ-DI and pain VAS reduction in the early stage of the treatment, which is a clue to prevention of a criteria of difficult-to-treat RA.

**Supplementary Information:**

The online version contains supplementary material available at 10.1186/s13075-024-03369-8.

## Introduction

Recent advances in the treatment of rheumatoid arthritis (RA) have dramatically improved the clinical and functional outcomes of the patients. A majority of RA patients now can achieve low disease activity (LDA) measured by parameters such as clinical disease activity index (CDAI), simplified disease activity index (SDAI), and disease activity score (DAS).

Nevertheless, some patients exhibit persistent symptoms even after achieving LDA, which is a part of the category of difficult-to-treat RA (D2T RA) by the European League Against Rheumatism (EULAR) [1] described as “well-controlled disease according to the above (universal) standards, but still having RA symptoms that are causing a reduction in quality of life”. A previous study reported that 35% of patients who achieved a moderate to good EULAR response did not consider their health to have improved a year after the treatment [2]. Another study showed that about one-fifth of patients with well-controlled disease activity do not feel well, determined subjectively [3], which can be a barrier to achieving the goal of treat-to-target.

Even though RA patients achieve LDA, persistent functional disability measured by high health assessment questionnaire-disability index (HAQ-DI) scores is often observed. In a previous cohort study, 10.9% of RA patients with persistently LDA had poor HAQ-DI scores [4]. Socioeconomic status, lifestyle, and social support [5] are also associated with higher HAQ-DI scores. However, these results might be partly due to less intensive treatment, which is often employed for patients with comorbidities and age-related physical dysfunction [6]. To exclude the contributions of differences in treatment intensity to disease activity measures, it is important to focus only on patients who are receiving sufficiently intensive treatment.

Here we analyzed the factors that are associated with the improvement of HAQ-DI and subjective pain of patients who achieved LDA by treatment with biological and targeted-synthetic (b/ts) disease-modifying antirheumatic drugs (DMARDs). The results of this study will inform the prevention of residual symptoms of RA patients and methods to optimize treat-to-target approaches.

## Methods

### Data source

The FIRST Registry is a multi-institutional cohort of RA patients treated with b/tsDMARDs, established by the University of Occupational and Environmental Health, Japan, and its multiple affiliated hospitals. Details of the cohort are available in other articles [7–10]. In this registry, all registered RA patients were enrolled in a long-term observational study at the time of receiving a new prescription or switching prescriptions of b/tsDMARDs. If a patient was treated with several b/tsDMARDs, each episode was treated as an independent episode.

By February 2023, 4842 patients were enrolled in the registry. In this study, b/tsDMARDs with the following four different mechanisms of action (classes) were included:

- Tumor necrosis factor inhibitors (TNFis): infliximab and its biosimilars, etanercept and its biosimilars, adalimumab and its biosimilars, golimumab, certolizumab pegol, and ozoralizumab.
- Interleukin-6 receptor inhibitors (IL-6Ris): tocilizumab, sarilumab, clazakizumab, and sirukumab.
- Cytotoxic T-lymphocyte–associated antigen-4 immunoglobulin (CTLA4-Ig): abatacept.
- Janus kinase inhibitors (JAKis): tofacitinib, baricitinib, peficitinib, and upadacitinib.

Rituximab was not included in this study because this drug was not yet approved by the Japanese government as a treatment option for RA. Clazakizumab and sirukumab were not included as new (current) prescription because they were still under testing.

At the start of b/tsDMARD treatment, baseline data were collected for all patients including demographics, disease duration, titers of rheumatoid factor (RF) and anti-cyclic citrullinated protein (anti-CCP) antibody, present and past treatments, serum creatinine levels, coexistence of interstitial lung disease (ILD), and past history of fractures and cancer. Measures of disease activity (CDAI, SDAI, DAS), functional status (HAQ-DI), duration of morning stiffness (MS), pain visual analogue scale (VAS), patients’ global health (GH), and evaluators’ global assessment (EGA), were also collected. Follow-up data on disease activity were collected at 6 months and one year after the start of therapy.

### Eligibility criteria

As the outcomes of treatment may differ when the treatment options are limited, this study included only patients who were enrolled in the FIRST Registry after JAKis were first approved in Japan, i.e., after August 2013. For the analysis of HAQ-DI improvement, patients with LDA (CDAI ≤ 10.0) at 6 months after starting treatment were included for further analyses.

### Exclusion criteria

Patients whose HAQ-DI data were not available at 6 months after starting treatment were excluded. To remove patients who received b/tsDMARDs as treatment for other autoimmune diseases (e.g., interstitial lung disease or vasculitis), patients treated with a > 15 mg/day prednisolone equivalent dose of glucocorticoids (GC) were excluded from the analysis. Patients who stopped treatment within 6 months were excluded for further analysis, but information about the reasons for the treatment cessation were collected.

### Definition of clinical and functional parameters

The following definitions were employed as indicators of clinical and functional improvement.

- Clinical LDA: CDAI ≤ 10.0 at 6 months.
- HAQ-DI normalization: HAQ-DI < 0.5 at 6 months, which is defined in a previous study [11].
- HAQ-DI improvement: improvement in HAQ-DI by > 0.22 units within 6 months, which is used in practice and in many other studies as a minimal clinically-important difference [4, 12–14].
- Pain VAS reduction: reduction in the pain VAS by ≥ 40 mm, which is considered to be a clinically relevant change [15], within 6 months.
- The changes in each clinical parameter within 6 months were calculated as follows:

Δvalue = (value at week 0) – (value at 6 months)

- Glucose intolerance: hemoglobin (Hb) A1c > 6.5% or fasting blood glucose of > 200 mg/dL at week 0.
- Overweight and obesity: body mass index (BMI) > 25 and > 30, respectively.
- Chronic kidney disease (CKD): Estimated glomerular filtration rate (eGFR) < 60 ml/min/1.73 m^2^. eGFR was calculated using the following formula:

$$\eqalign{{\rm{For}}\,{\rm{males:}}\,{\rm{194}}\,{\rm{ \times }}\,{\rm{serum}}&\,{\rm{creatinine}}\,{\left( {{\rm{mg/dL}}} \right)^{{\rm{ - 1}}{\rm{.094}}}}\,\cr&\quad{\rm{ \times }}\,{\rm{age}}\,{\left( {{\rm{years}}} \right)^{{\rm{ - 0}}{\rm{.287}}}}}$$

$$\eqalign{& {\rm{Fore}}\,{\rm{females:}}\,{\rm{194}}\,{\rm{ \times }}\,{\rm{serum}}\,{\rm{creatinine}} \cr & {\left( {{\rm{mg/dL}}} \right)^{{\rm{ - 1}}{\rm{.094}}}}\,{\rm{ \times }}\,{\rm{age}}\,{\left( {{\rm{years}}} \right)^{{\rm{- 0}}{\rm{.287}}}}\,{\rm{ \times }}\,{\rm{0}}{\rm{.739}} \cr}$$

### Statistical analysis

#### Simple comparison of patient background

The baseline data of the patients who achieved functional and pain improvement (HAQ-DI normalization, HAQ-DI improvement, and Pain VAS reduction) and the patients who did not were compared. The Student’s t-test was used for numerical variables and the chi-square test for categorical variables.

#### Multiple regression analysis

Factors associated with HAQ-DI normalization, HAQ-DI improvement, and pain VAS reduction at 6 months and one year after starting treatment were analyzed using multiple logistic regression. Sensitivity analyses were conducted including only the patients whose disease duration was < 5 years. Another sensitivity analyses was performed including only the patients who achieved CDAI remission (< 2.8).

As there are many factors that may confound each other, the degree of multicollinearity was detected by determining the variance inflation factor (VIF). A value of less than 5 indicated that the correlation was not severe enough to require modification.

To determine which clinical symptoms had the greatest impact on the improvement of HAQ-DI, the associations between the HAQ-DI improvement and the change in each clinical value i.g. pain VAS, tender joint count (TJC), swollen joint count (SJC), GH, duration of MS, titer of erythrocyte sedimentation rate (ESR), and EGA were analyzed by simple comparisons and multiple logistic regression.

These statistical analyses were carried out using Stata/SE 16.0 (StataCorp LLC, College Station, TX, USA). P-values of < 0.05 were considered to be statistically significant.

## Results

**Fig. 1:**
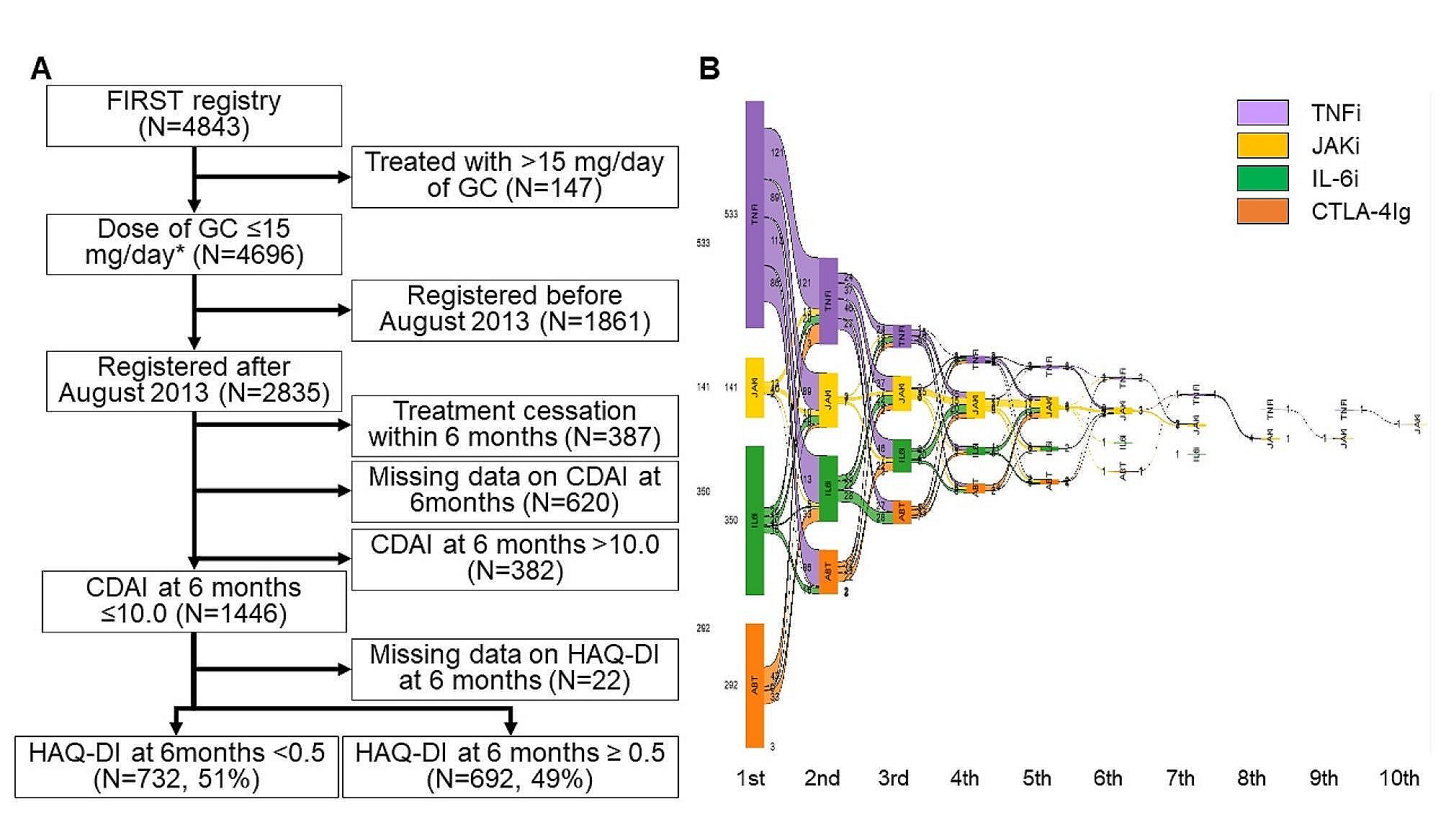
Process of patients’ selection. (**A**) Screening process. (**B**) Patterns of treatment switching

By February 2023, 4843 patients were enrolled in the FIRST Registry, of which 147 patients were treated with a > 15 mg/day prednisolone equivalent dose of GC. Another 1861 patients were enrolled before August 2013 when a JAK inhibitor was launched here and thus were excluded from this study. Within 6 months, 387 patients stopped treatment and the CDAI values at 6 months were missing for 620 patients. Among the remaining 1827 patients, 1474 achieved LDA (CDAI ≤ 10.0) at 6 months. The treatment outcomes of those who achieved LDA and those who did not are shown in Table 1. In total, 42.7% of patients (49.7% of patients with CDAI ≤ 10.0 and 13.1% of patients with CDAI > 10.0) achieved LDA. After excluding the patients whose HAQ-DI scores were not available (*N* = 22), 732 patients who achieved HAQ-DI < 0.5 and 692 patients who did not were included for further analyses (Fig. 1A). Patterns of treatment switching are shown in Fig. 1B.

To show the heterogeneity of patients by treatment types, the backgrounds and treatment outcomes and a breakdown of the reasons for treatment cessation by class of b/tsDMARDs were shown in Additional file 1 and Additional file 2, respectively. Although there was a significant heterogeneity in the background, the proportion of patients who achieved CDAI ≤ 10.0 did not significantly differ between drug types. Proportion of adverse events were small and did not seem to differ between drug types.

**Table 1:** Treatment outcomes at 6 months by disease activity

		Total (*N* = 1828)			Disease activity at 6 months					
					Remission/LDA (CDAI ≤ 10, *N* = 1446)			MDA/HDA (CDAI > 10, *N* = 382)		
		Mean	SE	Median	Mean	SE	Median	Mean	SE	Median
CDAI at 6 months		6.50	0.16	4.7	3.74	0.08	3.3	17.14	0.38	14.7
SDAI at 6 months		6.79	0.17	4.9	3.97	0.08	3.54	17.78	0.41	14.985
DAS28-ESR at 6 months		2.90	0.03	2.76	2.50	0.02	2.45	4.47	0.05	4.43
HAQ-DI at 6 months		0.78	0.02	0.625	0.64	0.02	0.375	1.33	0.04	1.25
Pain VAS at 6 months		28.00	0.57	21	21.18	0.52	15	53.99	1.19	57
Change in clinical parameters at 6 months	Δ CDAI	-18.33	0.29	-17	-19.80	0.31	-18	-12.66	0.67	-11.3
	Δ SDAI	-19.99	0.03	-18.34	-21.54	0.35	-19.38	-13.96	0.74	-12.51
	Δ DAS28-ESR	-2.46	0.03	-2.44	-2.77	0.04	-2.72	-1.30	0.07	-1.26
	Δ HAQ	-0.40	0.02	-0.25	-0.46	0.02	-0.25	-0.19	0.03	-0.125
	Δ Pain VAS	-23.70	0.67	-20	-27.63	0.73	-24	-8.67	1.35	-7.5
		N	%		N	%		N	%	
HAQ-DI improvement*		993	54.3		839	58.0		154	40.3	
HAQ-DI at 6 months ≤ 0.5		783	42.8		732	50.7		50	13.1	
Pain VAS reduction at 6month**		490	26.8		454	31.4		36	9.4	

SE: standard error, HAQ-DI: health assessment questionnaire disability index, CDAI: clinical disease activity index, SDAI: simplified disease activity index, DAS: disease activity score; VAS: visualized analogue scale, LDA: low disease activity, MDA: middle disease activity, HDA: high disease activity

*Those whose HAQ-DI improved by > 0.22, ** Those whose Pain VAS reduced by ≥ 40 mm

### Background of the patients

The background of the included patients is shown in Table 2. All factors except the mean BMI, the titers of RF and anti-CCP antibody, and the proportion of glucose intolerance showed significant differences between the HAQ-DI normalization and non-normalization groups.

**Table 2:** Background of the participants by HAQ-DI < 0.5 or ≥ 0.5 at 6 months. The two groups were compared using Student’s t-test for continuous variables and the chi-square test for categorical variables

		Total (*N* = 1424)			HAQ-DI < 0.5 at 6 month (*N* = 732)			HAQ-DI ≥ 0.5 at 6 month (*N* = 692)			*p*
Continuous variables		Mean	SE	Median	Mean	SE	Median	Mean	SE	Median	
Age		61.54	0.37	64	58.5	0.5	61	64.6	0.51	67	< 0.01
Disease duration (month)		98.19	2.96	57	74.3	3.2	42.5	121.8	4.96	78	< 0.01
BMI		22.40	0.10	21.9	22.44	0.13	22.0	22.40	0.16	21.8	0.20
eGFR		78.97	0.65	78.7	81.34	0.85	80.9	76.51	1.00	75.9	< 0.01
RF (IU/mL)		161.37	9.18	59.9	162.0	14.8	53.5	163.8	11.5	70.3	0.18
anti-CCP antibody (U/mL)		337.52	17.37	63.4	360.1	27.1	71.7	312.0	22.5	57.8	0.50
CDAI at week 0		23.55	0.31	21.7	21.39	0.44	19.35	25.78	0.45	24	< 0.01
SDAI at week 0		25.52	0.35	23.41	23.17	0.49	20.85	27.91	0.49	25.75	< 0.01
DAS28-ESR at week 0		5.27	0.02	5.26	4.98	0.05	4.94	5.56	0.05	5.52	< 0.01
HAQ-DI at week 0		1.11	0.02	1	0.73	0.02	0.5	1.49	0.03	1.38	< 0.01
Pain VAS at week 0		48.85	0.69	50	43.9	1.00	43.5	54.1	0.9	55	< 0.01
EGA at week 0		41.68	0.51	40	38.7	0.7	38	44.7	0.7	45	< 0.02
GH at week 0		48.12	0.65	50	43.1	0.94	45	53.3	0.9	52	< 0.03
Dose of MTX (mg/week)		8.78	0.16	10	9.62	0.23	12	8.00	0.24	8	< 0.01
Dose of GC (mg/day, PSL equivalent)		1.04	0.07	0	0.86	0.09	0	1.25	0.10	0	< 0.01
Categorical variables		N		%	N		%	N		%	p
Age	< 40	127		8.9	91		12.4	36		5.2	< 0.01
	40–49	153		10.7	96		13.1	57		8.2	
	50–59	273		19.2	153		20.9	120		17.3	
	60–69	393		27.6	207		28.3	186		26.9	
	70–79	380		26.7	163		22.3	217		31.4	
	≥ 80	98		6.9	22		3.0	76		11.0	
Female		1149		80.7	552		75.4	597		86.3	< 0.01
Disease duration	< 1y	292		20.5	185		25.3	107		15.5	< 0.01
	1-2y	170		11.9	84		11.5	86		12.4	
	2-5y	268		18.8	149		20.4	119		17.2	
	5-10y	262		18.4	147		20.1	115		16.6	
	10-20y	279		19.6	125		17.1	154		22.3	
	> 20y	153		10.7	42		5.7	111		16.0	
Overweight (BMI > 25)		305		21.4	145		19.8	160		23.1	0.13
Obesity (BMI > 30)		57		4.0	20		2.7	37		5.3	0.01
CKD		284		19.9	117		16.0	167		24.1	< 0.01
Glucose intolerance		133		9.3	58		7.9	75		10.8	0.06
ILD		302		21.2	124		16.9	178		25.7	< 0.01
Past history of fracture		227		15.9	87		11.9	140		20.2	< 0.01
Past history of cancer		170		11.9	74		10.1	96		13.9	0.03
≥ 2 classes of b/tsDMARD failure		479		33.6	190		26.0	289		41.8	< 0.01
≥ 2 csDMARD failure		227		15.9	99		13.5	128		18.5	0.01
Use of MTX at week 0		1033		72.5	562		76.8	471		68.1	< 0.01
Use of GC at week 0		270		19.0	109		14.9	161		23.3	< 0.01
RF positive (> 15 IU/mL)		1096		77.0	566		77.3	530		76.6	< 0.01
anti-CCP antibody positive (> 4.5 U/mL)		1019		71.6	529		72.3	490		70.8	< 0.01
Disease activity	LDA (CDAI ≤ 10)	133		9.3	101		13.8	32		4.6	< 0.01
	MDA (10 < CDAI ≤ 22)	592		41.6	324		44.3	268		38.7	
	HDA (22 < CDAI)	691		48.5	301		41.1	390		56.4	
Class of b/tsDMARD	TNFi	525		36.9	318		43.4	207		29.9	< 0.01
	IL6i	388		27.2	169		23.1	219		31.6	
	ABT	271		19.0	102		13.9	169		24.4	
	JAKi	240		16.9	143		19.5	97		14.0	
Use of MTX at week 0**		1033		72.5	562		76.8	471		68.1	< 0.01
Use of GC at week 0***		270		19.0	109		14.9	161		23.3	< 0.01

SE: standard error; HAQ-DI: health assessment questionnaire disability index, BMI: body mass index, GFR: glomerular filtration rate, RF: rheumatoid factor. CCP: cyclic citrullinated peptide, CDAI: clinical disease activity index, SDAI: simplified disease activity index, DAS: disease activity score; VAS: visualized analogue scale, EGA: evaluator’s global assessment, GH: patient’s global health, MTX: methotrexate, GC: glucocorticoid, CKD: chronic kidney disease, ILD: interstitial lung disease, b/tsDMARD: biological and targeted synthetic disease modifying anti-rheumatic drug, csDMARD: conventional synthetic disease modifying anti-rheumatic drug, LDA: low disease activity, MDA: middle disease activity, HDA: high disease activity, TNFi: tumor necrosis factor inhibitor, IL6i: interleukin-6 inhibitor, CTLA4-Ig: cytotoxic T-lymphocyte-associated antigen 4 immunoglobulin, JAKi: Janus kinase inhibitor

### Pre-treatment factors associated with HAQ-DI normalization (< 0.5) at 6 months

**Fig. 2:**
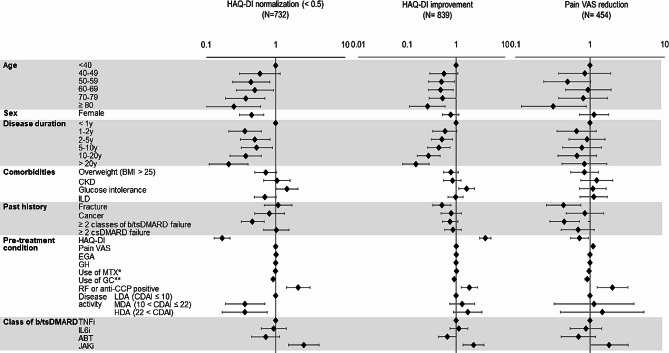
Multiple logistic regression analyses for factors related to HAQ-DI normalization (left column), HAQ-DI normalization (middle column), and pain VAS reduction (right column)

Multiple logistic regression analysis was conducted to identify the factors associated with HAQ-DI normalization (< 0.5) at 6 months (Fig. 2, left column and Additional file 3). Age, sex, disease duration, coexisting diseases, refractory status to past DMARDs, RA-related status at week 0, the class of b/tsDMARD, and the concomitant use of methotrexate (MTX) and GC were employed as explanatory factors in this analysis. The seropositivity of RF or anti-CCP antibody [odds ratio (OR) 2.10, 95% confidence interval (CI) 1.42–3.12] and the use of JAKi compared with the use of TNFi (OR 2.52, 95%CI 1.52–4.18) were associated with the normalization. On the other hand, older age, longer disease duration, being female (OR 0.45, 95% CI 0.30–0.67), past failure in ≥ 2 classes of b/tsDMARDs (OR 0.46, 95% CI 0.32–0.68), the use of GC (OR 0.92, 95% CI 0.86–0.99), higher HAQ-DI at week 0 (OR 0.17, 95% CI 0.13–0.22), MDA and HDA at week 0 compared with LDA (OR 0.36, 95% CI 0.19–0.70 and OR 0.36, 95% CI 0.17–0.75, respectively) were associated with a lower likelihood of HAQ-DI normalization. The same analysis was conducted with outcome of HAQ-DI normalization at 1 year (Additional file 4). Similar tendency was observed, though the association was no more significant in the use of JAKi and GC.

Sensitivity analyses were applied to the patients with a disease duration of < 5 years (Additional file 5) and the who achieved CDAI remission (< 2.8) at 6 months (Additional files 6). Older age, longer disease duration, being female and past failure in ≥ 2 classes of b/tsDMARDs remained to be associated with lower likelihood of HAQ-DI improvement. However, the association was no more significant in the use of GC.

As the differences in HAQ-DI between patients with seropositivity or seronegativity of the RF and anti-CCP may reflect differences in the background of the patients, the backgrounds and outcome values were compared between seropositive and seronegative patients (Additional file 7). Seropositive patients were of older age, with longer disease durations, lower BMI values, higher eGFR values, and lower doses of GC compared with seronegative patients. On average, the seropositive patients showed higher HAQ-DI and DAS28-ESR scores at 6 months. However, the proportion of pain VAS reduction was also higher among the seropositive patients.

### Factors associated with HAQ-DI improvement

HAQ-DI normalization at 6 months may have been due to low HAQ-DI scores at the start of the treatment, the factors associated with a significant improvement in HAQ-DI (by ≥ 0.22 units) were also analyzed (Fig. 2, middle column and Additional file 8). In total, 839 patients (59%) showed improvement of HAQ-DI scores. Multiple logistic regression analysis showed that seropositivity of RF and anti-CCP antibody (OR 1.86, 95% CI 1.27–2.72), higher HAQ-DI at baseline (OR 3.93, 95% CI 3.01–5.15), and the use of JAKi compared with the use of TNFi (OR 2.26, 95% CI 1.38 − 3.71) were associated with HAQ-DI improvement. On the other hand, an age of ≥ 80 years compared with an age of < 40 years (OR 0.26, 95% CI 0.11–0.59), a longer disease duration, a past history of fracture (OR 0.51, 95% CI 0.33–0.78), and the use of GC (OR 0.91, 95% CI 0.85–0.97) were associated with a lower likelihood of HAQ-DI improvement.

The proportion of patients with HAQ-DI improvement, among those who achieved LDA, might be affected by the proportion of LDA achievement with each drug class or each age group. However, the proportions of patients with LDA versus MDA-HDA at 6 months were not significantly different between the treatment classes (Additional file 1) and age groups (Additional file 9).

### Factors associated with pain VAS reduction

Among the 1424 patients who achieved LDA, 454 (31.8%) achieved pain VAS reduction (≥ 40 mm) at 6 months. Multiple logistic regression analysis was conducted to identify the factors associated with pain VAS reduction (Fig. 2, right column and Additional file 10). Seropositivity of RF or anti-CCP antibody (OR 2.00, 95% CI 1.25 − 3.22), the use of JAKi compared with the use of TNFi (OR 1.80, 95% CI 1.00–3.24), and higher pain VAS scores at baseline (OR 1.10, 95% CI 1.09–1.12 for a 1 mm increase in pain VAS) were associated with pain VAS reduction. On the other hand, an age of ≥ 80 years compared with an age of < 40 years (OR 0.32, 95% CI 0.12–0.89), a past history of fracture (OR 0.44, 95% CI 0.26–0.75), the failure of ≥ 2 classes of b/tsDMARD (OR 0.45, 95% CI 0.29–0.72), the use of GC (OR 0.92, 95% CI 0.85–0.99), and a higher HAQ-DI score at baseline (OR 0.72, 95%CI 0.55–0.95) were associated with a lower likelihood of pain VAS reduction. The same analysis was conducted with outcome of pain VAS reduction at 1 year (Additional file 11). In this phase, only higher pain VAS at baseline and the failure of ≥ 2 classes of b/tsDMARD were associated with a lower likelihood of the reduction.

For sensitivity analyses, the same regression models used for Additional file 10 were applied to the patients with a disease duration of < 5 years (Additional file 12) and the who achieved CDAI remission (< 2.8) (Additional file 13). The similar tendency was observed, though the association with the use of GC was no more observed.

### Post-treatment factors associated with HAQ-DI improvement

As HAQ-DI improvement status is affected by a variety of clinical symptoms, we analyzed the association between the changes in clinical variables (pain VAS, TJC, SJC, GH, MS, ESR, EGA) and HAQ-DI improvement (Table 3). All clinical variables improved more among the HAQ-DI improvement group compared with the non-improvement group. When logistic regression was conducted, improvements in the pain VAS, GH, and ESR were significantly associated with HAQ-DI improvement.

**Table 3:** Change in clinical variables within 6 months by HAQ-DI improved (> 0.22) or not

	Total (*N* = 1474)			HAQ-DI improvement (*N* = 839, 57%)			HAQ-DI non-improvement (*N* = 635, 43%)			p*	OR	95%CI		p**
	Mean	SE	Median	Mean	SE	Median	Mean	SE	Median					
Δ Pain VAS	-27.63	0.73	-24	-36.62	0.94	-35	-14.70	0.96	-11	< 0.01	0.98	0.97	0.99	< 0.01
Δ TJC	-9.47	0.20	-8	-10.89	0.28	-9	-7.46	0.29	-6	< 0.01	0.98	0.96	1.00	0.08
Δ SJC	-8.07	0.17	-7	-9.39	0.24	-8	-6.23	0.22	-5	< 0.01	0.98	0.95	1.01	0.15
Δ GH	-25.07	0.69	-22	-32.42	0.93	-32	-13.64	0.91	-10	< 0.01	0.99	0.98	1.00	0.02
Δ MS	-97.27	10.15	-25	-128.87	13.43	-50	-61.01	15.86	0	< 0.01	1.00	1.00	1.00	0.53
Δ ESR	-25.74	0.77	-20	-31.59	1.05	-26	-17.33	1.07	-12	< 0.01	0.99	0.98	1.00	< 0.01
Δ EGA	-35.90	0.53	-35	-40.53	0.71	-40	-28.99	0.77	-29	< 0.01	0.99	0.98	1.00	0.05

HAQ-DI: health assessment questionnaire disability index, VAS: visualised analogue scale, TJC: tender joint counts, SJC: swollen joint counts, GH: patient’s global health, MS: morning stiffness, ESR: erythrocyte sedimentation rate, EGA: evaluator’s global assessment

*simple comparison using Student’s t-test

** logistic regression test using all the variables in the table

To detect collinearity of the explanatory factors, the VIF was calculated (Additional file 14). No factor showed a VIF > 5 and thus all factors were included in the analysis.

## Discussion

This study analyzed the factors associated with improvement of HAQ-DI and reduction of subjective pain among RA patients who achieved LDA within 6 months and one year of treatment. The results showed that the pretreatment background and treatment options were both associated with the improvement. According to EULAR, the situation in which a patient has “well-controlled disease according to the above (universal) standards, but still having RA symptoms that are causing a reduction in quality of life” is categorized as one of the criteria of D2T RA [1]. As this is the flipside of the outcome of our study (improvement in symptoms after achieving LDA), our results may provide insights regarding the prediction of D2T RA to some extent.

A previous study targeting early RA patients showed that patients in the “low inflammation - high HAQ” group were on average older, were more often female, had more comorbidities and had more severe pain, fatigue, anxiety and depressive symptoms at baseline compared with patients in the “low inflammation - low HAQ” group [16]. Similarly, previous studies of RA patients have shown an association between comorbidities and poor functional outcomes [17] and difficulty in disease control [18]. These poor outcomes were partly attributed to the limited treatment options among patients with comorbidities [19]. However, our study showed that existing comorbidities such as overweight, CKD, and ILD, and a past history of cancer were not associated with HAQ-DI normalization. This is consistent with previous studies showing that comorbidities may not affect the refractoriness of treatment [20] or the improvement in physical function when controlling for other factors [21]. This discrepancy can be explained by the current development of b/tsDMARDs with better safety profiles, which made it possible for patients with comorbidities to receive more intensive treatment than in the past. Indeed, previous study suggested that significant increase in mortality rate among RA patients might be eliminated when they are treated with bDMARD [22]. Given these results, introduction of b/tsDMARDs in the early phase of RA would be recommended especially for patients with complications such as ILD and CKD.

On the other hand, higher age correlated with poor improvements in HAQ-DI and pain VAS scores (Fig. 2). As the proportion of LDA achievement was not different between age groups (Additional file 9), this result may reflect an increase in the baseline of pain VAS with age.

Considering each clinical factor, HAQ-DI improvement is strongly associated with the reduction of pain VAS rather than the number of tender joints or swollen joints (Table 3). Therefore, physicians may need to consider treatment intensification for patients with high pain levels, even after the number of affected joints has decreased. Interestingly, patients who experienced the failure of ≥ 2 classes of b/tsDMARD were less likely to achieve HAQ-DI normalization and pain VAS reduction. However, our previous study showed that the failure of ≥ 2 b/tsDMARDs does not predict poor CDAI improvement in response to b/tsDMARDs [23]. Therefore, the major cause of the D2T status of these patients might be due to residual pain rather than inflammatory status.

Our results also showed a negative correlation between HAQ-DI improvement / pain VAS reduction and the use of GC. Since a past history of fractures is also associated with these outcomes, this might be caused by steroid-induced osteoporosis and fractures. Although further prospective research is required, the early introduction of rapid-acting b/tsDMARDs rather than GC may be a preferable option for patients with a high risk of osteoporosis [24]. Another hypothesis is that inflammatory symptom such as swollen joints is masked among patients using GC who did not actually achieved LDA. This is supported by the result that the difference was no more observed when only those who achieved remission were included (Additional file 6, 13). In addition, promoted catabolism and suppression of hypothalamus-pituitary-adrenal axis caused by long-term GC treatment may increase risks of fatigue and malaise and thus affect physical activities. Further research including change in hormonal status may be needed to understand the mechanisms of residual pain among patients who achieve LDA.

In our study, JAKi were associated with HAQ-DI normalization and pain VAS reduction compared with TNFi at 6 months after starting treatment. The effectiveness of JAKi on patient-oriented outcomes among RA patients are well-established [25], including the effectiveness on pain among RA patients with low levels of inflammation [26]. This might be due to the direct effect of JAKi on signals on pain sensitivity [27], or the rapid-acting nature of these agents, within 24 h [24], one week [28], and two weeks [10], which may also contribute to the improvements in HAQ-DI in the early stages of treatment. This assumption is supported by the observation that the difference between drug classes was not significant one year after starting treatment (Additional file 4, 11). As the proportions of patients who achieved LDA were not significantly different (Additional file 1), this difference is less likely to be caused by the difference in response rates to the treatment at 6 months. Even so, this difference might be due to the differences in pre-treatment conditions that were not included in this study, and therefore, we should be careful in the interpretation of this result.

Interestingly, there was positive relationship between seropositivity of RF / anti-CCP antibodies and the improvement in clinical symptoms. Previous research associate seropositivity of ACPA and higher disease activity [29]. RF positivity with positive anti-CCP antibodies was also associated with higher systemic inflammation in early RA [30]. As the effect is observed when we included only those who achieved LDA or remission (Additional file 6 and 13), the difference was less likely to be persistent inflammation of the patients. Another possibility is difference in background between seropositive and seronegative patients (Additional file 7). Especially, disease duration is longer among seropositive patients. However, the difference remained significant even among those with shorter disease duration (< 5 years, Additional file 5, 12). Another possibility is that seropositive patients might have been diagnosed as b/tsDMARDs earlier compared with seronegative patients. In addition, promotion of osteoclastogenesis and nociception by autoantibodies [31] might be cancelled by earlier intensive treatment with b/tsDMARDs [22] that improves HAQ-DI and Pain VAS.

### Limitations

Our study has several limitations, primarily due to its retrospective nature. First, this registry included several episodes of treatments of the same patients with different agents. Second, other comorbidities that may confound the outcomes, such as hepatic disorders and neurological disorders, were not included. Information about the severity of ILD was not collected, suggesting that our data about pre-existing comorbidities may not have been sufficient to determine a possible association. Third, some treatment options such as rituximab were not included in our study because it is not approved as a treatment for RA in Japan. In addition, psychological factors such as SF-36 scores were not included in this analysis, and the impact of these factors on functional outcomes is not clear. For example, a negative correlation between pain VAS reduction and longer disease duration may partly be caused by an increase in the proportion of patients with depressive status or fibromyalgia with time. Further research is required, including the use of drugs such as antidepressants and pregabalin for neuropathic pain. Nevertheless, our study is important in that it provides a clue to risk factors and beneficial factors affecting D2T RA.

## Conclusions

Our study revealed several factors that are associated with a category of D2T RA, “well-controlled disease according to the above (universal) standards, but still having RA symptoms that are causing a reduction in quality of life”. Longer disease duration and a past history of fracture were associated with less improvement in HAQ-DI and pain VAS, suggesting the importance of T2T even among patients who achieve LDA in the early phases of treatment. As the failure of ≥ 2 classes of b/tsDMARD was associated with poor pain VAS reduction, further research might be required for the prevention of D2T RA. Our study also suggested that GC use is not preferable with regard to HAQ-DI and pain improvement. Therefore, the use of rapid-acting b/tsDMARDs instead of GC might be a preferable treatment option for patients with high disease activity, especially when the patients have osteoporosis.

### Electronic supplementary material

Below is the link to the electronic supplementary material.

Supplementary Material 1

## Data Availability

The datasets used and/or analyzed during the current study are available from the corresponding author on reasonable request.

## Abbreviations

BMI: Body mass index
b/tsDMARD: Biological and targeted synthetic disease modifying anti-rheumatic drug
csDMARD: Conventional synthetic disease modifying anti-rheumatic drug
CCP: Cyclic citrullinated peptide
CDAI: Clinical disease activity index
CI: Confidence interval
CKD: Chronic kidney disease
CTLA4-Ig: Cytotoxic T-lymphocyte-associated antigen 4 immunoglobulin
D2T RA: Difficult-to-treat rheumatoid arthritis
DAS: Disease activity score
EGA: Evaluator’s global assessment
ESR: Erythrocyte sedimentation rate
GC: Glucocorticoid
GFR: Glomerular filtration rate
GH: Patient’s global health
HAQ-DI: Health assessment questionnaire disability index
HDA: High disease activity
IL6i: Interleukin-6 receptor inhibitor
ILD: Interstitial lung disease
JAKi: Janus kinase inhibitor
LDA: Low disease activity
MDA: Middle disease activity
MS: Morning stiffness
MTX: Methotrexate
OR: Odds ratio
RA: Rheumatoid arthritis
RF: Rheumatoid factor
SDAI: Simplified disease activity index
SJC: Swollen joints count
TJC: Tender joints count
TNFi: Tumor necrosis factor inhibitor
VAS: Visualized analogue scale
