# Supplementary material for: Predictors of functional improvement and pain reduction in rheumatoid arthritis patients who achieved low disease activity with disease-modifying antirheumatic drugs: a retrospective study of the FIRST Registry
Source: Arthritis Res Ther. 2024 Jul 26;26:140. doi: 10.1186/s13075-024-03369-8 (PMC11282705; doi:10.1186/s13075-024-03369-8)
Supplement: Supplementary file 1 — Supplementary Material 1 [file 13075_2024_3369_MOESM1_ESM.docx]

**Additional file 1. Background and outcomes of the participants by classes of b/tsDMARD.**

|  | | TNFi　(N = 986) | | | IL-6i　(N = 805) | | | CTLA4-Ig　(N = 556) | | | JAKi 　(N = 465) | | | p |
| --- | --- | --- | --- | --- | --- | --- | --- | --- | --- | --- | --- | --- | --- | --- |
| **Background** | | Mean | SE | Median | Mean | SE | Median | Mean | SE | Median | Mean | SE | Median |  |
| Age | | 58.56 | 0.52 | 60 | 64.54 | 0.48 | 67 | 68.61 | 0.57 | 71 | 58.55 | 0.65 | 60 | <0.01 |
| Disease duration (month) | | 81.90 | 3.67 | 37 | 111.12 | 4.96 | 60 | 125.14 | 5.72 | 86.5 | 122.49 | 5.54 | 93.5 | <0.01 |
| BMI | | 22.51 | 0.14 | 21.97 | 22.72 | 0.15 | 22.20 | 22.33 | 0.20 | 21.80 | 22.78 | 0.20 | 22.21 | 0.22 |
| eGFR | | 84.54 | 0.85 | 83.14 | 72.59 | 1.03 | 71.80 | 70.02 | 1.05 | 69.43 | 80.72 | 1.17 | 80.20 | <0.01 |
| RF (IU/mL) | | 138.32 | 11.65 | 51.3 | 191.01 | 15.44 | 56.7 | 226.82 | 30.60 | 88.65 | 213.66 | 22.91 | 63.6 | <0.01 |
| anti-CCP antibody (U/mL) | | 310.97 | 20.41 | 59.8 | 354.94 | 32.53 | 56.5 | 407.66 | 39.22 | 111.3 | 320.45 | 31.86 | 56.3 | <0.01 |
| CDAI at week 0 | | 24.32 | 0.45 | 22.7 | 25.17 | 0.48 | 23.3 | 23.69 | 0.51 | 22.25 | 24.80 | 0.58 | 23.6 | 0.01 |
| SDAI at week 0 | | 25.92 | 0.49 | 24.04 | 28.08 | 0.54 | 25.36 | 25.20 | 0.53 | 24.06 | 26.13 | 0.63 | 24.36 | <0.01 |
| DAS28-ESR at week 0 | | 5.23 | 0.05 | 5.24 | 5.58 | 0.05 | 5.61 | 5.34 | 0.05 | 5.37 | 5.16 | 0.06 | 5.18 | 0.04 |
| HAQ-DI at week 0 | | 1.07 | 0.03 | 1 | 1.27 | 0.03 | 1.125 | 1.31 | 0.04 | 1.25 | 1.20 | 0.04 | 1.125 | 0.35 |
| Pain VAS at week 0 | | 49.78 | 0.91 | 50 | 52.90 | 1.01 | 53 | 51.55 | 1.15 | 51 | 52.06 | 1.28 | 55 | 0.47 |
| EGA at week 0 | | 41.55 | 0.70 | 40 | 44.82 | 0.77 | 45 | 41.55 | 0.87 | 40 | 45.07 | 1.05 | 45 | 0.09 |
| Global Health at week 0 | | 48.37 | 0.87 | 50 | 52.10 | 0.94 | 51 | 50.68 | 1.10 | 50 | 52.59 | 1.22 | 56 | 0.89 |
| Dose of MTX (mg/week) | | 11.00 | 0.18 | 12 | 5.75 | 0.23 | 4 | 6.75 | 0.29 | 6 | 8.33 | 0.31 | 10 | <0.01 |
| Dose of GC (mg/day, PSL equivalent) | | 0.57 | 0.06 | 0 | 1.58 | 0.12 | 0 | 1.25 | 0.12 | 0 | 0.89 | 0.11 | 0 | <0.01 |
| **Outcome** | |  |  |  |  |  |  |  |  |  |  |  |  |  |
| CDAI at 6 months | | 5.89 | 0.26 | 4 | 6.75 | 0.30 | 5 | 7.47 | 0.38 | 5.65 | 6.24 | 0.39 | 4.2 | 0.14 |
| SDAI at 6 months | | 6.15 | 0.26 | 4.36 | 6.86 | 0.32 | 5 | 8.08 | 0.40 | 6.165 | 6.53 | 0.40 | 4.62 | 0.04 |
| DAS28-ESR at 6 months | | 2.87 | 0.04 | 2.69 | 2.49 | 0.05 | 2.28 | 3.45 | 0.06 | 3.325 | 3.04 | 0.06 | 2.845 | 0.99 |
| HAQ at 6 months | | 0.62 | 0.03 | 0.375 | 0.83 | 0.03 | 0.6875 | 1.06 | 0.05 | 1 | 0.72 | 0.05 | 0.5 | <0.01 |
| Pain VAS at 6 months | | 25.36 | 0.92 | 18 | 29.22 | 1.09 | 22 | 31.90 | 1.32 | 25 | 26.96 | 1.37 | 20 | 0.56 |
| Change in clinical parameters | Δ CDAI | -18.64 | 0.49 | -16.65 | -18.68 | 0.55 | -17.5 | -16.65 | 0.61 | -14.85 | -19.08 | 0.75 | -18.2 | 0.22 |
|  | Δ SDAI | -20.01 | 0.06 | -17.62 | -21.54 | 0.06 | -20.08 | -17.52 | 0.07 | -15.705 | -20.31 | 0.09 | -19.09 | 0.01 |
|  | Δ DAS28-ESR | -2.40 | 0.06 | -2.36 | -3.11 | 0.06 | -3.17 | -1.93 | 0.07 | -1.87 | -2.17 | 0.09 | -2.12 | 0.06 |
|  | Δ HAQ | -0.45 | 0.02 | -0.25 | -0.40 | 0.03 | -0.25 | -0.27 | 0.03 | -0.125 | -0.48 | 0.04 | -0.375 | 0.32 |
|  | Δ Pain VAS | -24.48 | 1.06 | -21 | -24.26 | 1.36 | -22 | -20.73 | 1.43 | -19 | -24.65 | 1.63 | -18 | 0.02 |
|  |  | **N** | **%** |  | **N** | **%** |  | **N** | **%** |  | **N** | **%** |  |  |
| Disease activity | CDAI ≤ 10 | 126 | 18.9 |  | 108 | 21.1 |  | 85 | 23.2 |  | 63 | 19.0 |  | 0.43 |
|  | 10 < CDAI | 540 | 81.1 |  | 405 | 78.9 |  | 281 | 76.8 |  | 248 | 74.9 |  |  |
| HAQ-DI improvement | | 375 | 56.3 |  | 272 | 53.0 |  | 169 | 46.2 |  | 192 | 58.0 |  | 0.02 |
| HAQ-DI at 6 month ≤ 0.5 | | 342 | 51.4 |  | 189 | 36.8 |  | 110 | 30.1 |  | 152 | 45.9 |  | <0.01 |
| Pain VAS reduction at 6months | | 174 | 26.1 |  | 145 | 28.3 |  | 84 | 23.0 |  | 93 | 28.1 |  | 0.19 |

HAQ-DI: health assessment questionnaire disability index, BMI: body mass index, GFR: glomerular filtration rate, RF: rheumatoid factor. CCP: cyclic citrullinated peptide, CDAI: clinical disease activity index, SDAI: simplified disease activity index, DAS: disease activity score; VAS: visualised analogue scale, EGA: evaluator’s global assessment, MTX: methotrexate, GC: glucocorticoid, TNFi: tumour necrosis factor inhibitor, IL6i: interleukin-6 inhibitor, CTLA4-Ig: cytotoxic T-lymphocyte-associated antigen 4 immunoglobulin, JAKi: Janus kinase inhibitor

**Additional file 2. Breakdown of those who stopped treatment within 6 months by classes of b/tsDMARDs.**

|  | TNFi  (N = 986) | | IL-6i  (N = 805) | | CTLA4-Ig  (N = 556) | | JAKi  (N = 465) | | Total  (N = 2813) | |
| --- | --- | --- | --- | --- | --- | --- | --- | --- | --- | --- |
| Treatment cessation (N, %) | 162, 16.4% | | 92, 11.4%) | | 69, 12.4% | | 42, 9.0% | | 365, 13.0% | |
|  | N | % | N | % | N | % | N | % | N | % |
| Primary failure | 61 | 37.7 | 21 | 22.8 | 28 | 40.6 | 6 | 14.3 | 116 | 31.8 |
| Secondary failure | 27 | 16.7 | 6 | 6.5 | 9 | 13.0 | 5 | 11.9 | 47 | 12.9 |
| Insufficient effect | 15 | 9.3 | 14 | 15.2 | 13 | 18.8 | 8 | 19.0 | 50 | 13.7 |
| Remission | 6 | 3.7 | 2 | 2.2 | 2 | 2.9 | 0 | 0.0 | 10 | 2.7 |
| Malignancy | 4 | 2.5 | 0 | 0.0 | 0 | 0.0 | 1 | 2.4 | 5 | 1.4 |
| LPD | 0 | 0.0 | 2 | 2.2 | 0 | 0.0 | 2 | 4.8 | 4 | 1.1 |
| Other adverse events | 39 | 24.1 | 33 | 35.9 | 14 | 20.3 | 16 | 38.1 | 102 | 27.9 |
| Economic reason | 4 | 2.5 | 1 | 1.1 | 0 | 0.0 | 1 | 2.4 | 6 | 1.6 |
| Pregnancy | 0 | 0.0 | 1 | 1.1 | 0 | 0.0 | 1 | 2.4 | 2 | 0.5 |
| Non-adherence | 3 | 1.9 | 5 | 5.4 | 2 | 2.9 | 2 | 4.8 | 12 | 3.3 |
| Loss of follow up | 1 | 0.6 | 1 | 1.1 | 0 | 0.0 | 0 | 0.0 | 2 | 0.5 |
| Other | 2 | 1.2 | 6 | 6.5 | 1 | 1.4 | 0 | 0.0 | 9 | 2.5 |
| Total | 162 | 100 | 92 | 100 | 69 | 100 | 42 | 100 | 365 | 100 |

b/tsDMARD: biological and targeted synthetic disease modifying anti-rheumatic drug, TNFi: tumour necrosis factor inhibitor, IL6i: interleukin-6 inhibitor, CTLA4-Ig: cytotoxic T-lymphocyte-associated antigen 4 immunoglobulin, JAKi: Janus kinase inhibitor , LPD: lympho-proliferative diseases

**Additional file 3. Multiple logistic regression analysis for factors related to HAQ-DI normalization (< 0.5) at 6 months.**

|  | | | HAQ normalization (< 0.5)  (N=732) | | | |
| --- | --- | --- | --- | --- | --- | --- |
|  |  |  | OR | 95%CI | | p |
| Age | <40 | | 1 (Reference) | | | |
|  | 40-49 | | 0.59 | 0.30 | 1.16 | 0.13 |
|  | 50-59 | | 0.44 | 0.24 | 0.82 | 0.01 |
|  | 60-69 | | 0.50 | 0.27 | 0.93 | 0.03 |
|  | 70-79 | | 0.37 | 0.19 | 0.70 | <0.01 |
|  | ≥ 80 | | 0.25 | 0.10 | 0.60 | <0.01 |
| Female | | | 0.45 | 0.30 | 0.67 | <0.01 |
| Disease duration | <1y | | 1 (Reference) | | | |
|  | 1-2y | | 0.36 | 0.21 | 0.62 | 0.00 |
|  | 2-5y | | 0.50 | 0.31 | 0.83 | 0.01 |
|  | 5-10y | | 0.53 | 0.32 | 0.90 | 0.02 |
|  | 10-20y | | 0.37 | 0.22 | 0.62 | <0.01 |
|  | > 20y | | 0.21 | 0.11 | 0.40 | <0.01 |
| Comorbidities | Overweight (BMI > 25) | | 0.72 | 0.50 | 1.03 | 0.08 |
|  | CKD | | 1.04 | 0.67 | 1.61 | 0.86 |
|  | Glucose intolerance | | 1.45 | 1.01 | 2.08 | 0.04 |
|  | ILD | | 0.70 | 0.49 | 1.01 | 0.05 |
| Past history | Past history of fracture | | 1.08 | 0.68 | 1.71 | 0.74 |
|  | Past history of cancer | | 0.81 | 0.50 | 1.33 | 0.40 |
|  | ≥ 2 classes of b/tsDMARD failure | | 0.46 | 0.32 | 0.68 | <0.01 |
|  | ≥ 2 csDMARD failure | | 1.02 | 0.67 | 1.55 | 0.93 |
| Pre-treatment condition | HAQ-DI | | 0.17 | 0.13 | 0.22 | <0.01 |
|  | Pain VAS | | 1.00 | 0.99 | 1.01 | 0.50 |
|  | EGA | | 1.01 | 1.00 | 1.02 | 0.05 |
|  | GH | | 1.00 | 0.99 | 1.01 | 0.89 |
|  | Use of MTX | | 0.99 | 0.96 | 1.02 | 0.40 |
|  | Use of GC | | 0.92 | 0.86 | 0.99 | 0.02 |
|  | RF or anti-CCP positive | | 2.10 | 1.42 | 3.12 | <0.01 |
|  | Disease activity | LDA (CDAI ≤ 10) | 1 (Reference) | | | |
|  |  | MDA (10 < CDAI ≤ 22) | 0.36 | 0.19 | 0.70 | <0.01 |
|  |  | HDA (22 < CDAI) | 0.36 | 0.17 | 0.75 | 0.01 |
| Class of b/tsDMARD | TNFi | | 1 (Reference) | | | |
|  | IL6i | | 0.93 | 0.62 | 1.41 | 0.74 |
|  | ABT | | 0.72 | 0.45 | 1.13 | 0.15 |
|  | JAKi | | 2.52 | 1.52 | 4.18 | <0.01 |

b/tsDMARD: biological and targeted synthetic disease modifying anti-rheumatic drug, RF: rheumatoid factor. CCP: cyclic citrullinated peptide, CKD: chronic kidney disease, ILD: interstitial lung disease, csDMARD: conventional synthetic disease modifying anti-rheumatic drug, HAQ-DI: health assessment questionnaire disability index, VAS: visualized analogue scale, EGA: evaluator’s global assessment, GH: patient’s global health, CDAI: clinical disease activity index, LDA: low disease activity, MDA: middle disease activity, HDA: high disease activity, TNFi: tumor necrosis factor inhibitor, IL6i: interleukin-6 inhibitor, CTLA4-Ig: cytotoxic T-lymphocyte-associated antigen 4 immunoglobulin, JAKi: Janus kinase inhibitor, MTX: methotrexate, GC: glucocorticoid, OR: odds ratio, CI: confidence interval

**Additional file 4. Multiple regression analysis for factor related to HAQ-DI normalization (< 0.5) at 1 year of b/tsDMARD treatment.**

|  |  | |  | HAQ-DI < 0.5  at 1 year  (N = 705) | |  |
| --- | --- | --- | --- | --- | --- | --- |
|  |  | | OR | 95%CI | | p |
| Age | < 40 | | 1 (Reference) | | | |
|  | 40-49 | | 0.45 | 0.21 | 0.96 | 0.04 |
|  | 50-59 | | 0.36 | 0.18 | 0.73 | 0.01 |
|  | 60-69 | | 0.54 | 0.26 | 1.09 | 0.09 |
|  | 70-79 | | 0.36 | 0.18 | 0.76 | 0.01 |
|  | ≥ 80 | | 0.23 | 0.08 | 0.60 | <0.01 |
| Female | | | 0.36 | 0.23 | 0.55 | <0.01 |
| Disease duration | < 1y | | 1 (Reference) | | | |
|  | 1-2y | | 0.53 | 0.30 | 0.92 | 0.03 |
|  | 2-5y | | 0.48 | 0.29 | 0.80 | 0.01 |
|  | 5-10y | | 0.66 | 0.38 | 1.14 | 0.14 |
|  | 10-20y | | 0.45 | 0.26 | 0.76 | <0.01 |
|  | > 20y | | 0.22 | 0.12 | 0.43 | <0.01 |
| Comorbidities | Overweight (BMI > 25) | | 0.78 | 0.53 | 1.15 | 0.20 |
|  | CKD | | 0.77 | 0.50 | 1.19 | 0.24 |
|  | Glucose intolerance | | 1.12 | 0.77 | 1.61 | 0.56 |
|  | ILD | | 0.60 | 0.41 | 0.87 | 0.01 |
| Past history | Fracture | | 1.12 | 0.71 | 1.77 | 0.63 |
|  | Cancer | | 0.76 | 0.46 | 1.26 | 0.29 |
|  | ≥ 2 classes of b/tsDMARD failure | | 0.52 | 0.35 | 0.76 | <0.01 |
|  | ≥ 2 csDMARD failure | | 1.01 | 0.66 | 1.56 | 0.96 |
| Pre-treatment condition | HAQ-DI | | 0.20 | 0.15 | 0.26 | <0.01 |
|  | Pain VAS | | 1.00 | 0.99 | 1.01 | 0.55 |
|  | EGA | | 1.01 | 1.00 | 1.02 | 0.26 |
|  | Global Health | | 1.00 | 0.99 | 1.01 | 0.94 |
|  | Dose of MTX | | 1.21 | 0.79 | 1.85 | 0.39 |
|  | Dose of GC | | 0.72 | 0.45 | 1.14 | 0.16 |
|  | RF or anti-CCP positive | | 1.37 | 0.90 | 2.07 | 0.14 |
|  | Disease activity | LDA (CDAI ≤ 10) | 1 (Reference) | | | |
|  |  | MDA (10 < CDAI ≤ 22) | 0.64 | 0.33 | 1.24 | 0.19 |
|  |  | HDA (22 < CDAI) | 0.71 | 0.34 | 1.49 | 0.37 |
| Class of b/tsDMARD | TNFi | | 1 (Reference) | | | |
|  | IL6i | | 1.21 | 0.79 | 1.85 | 0.39 |
|  | ABT | | 0.72 | 0.45 | 1.14 | 0.16 |
|  | JAKi | | 1.56 | 0.94 | 2.59 | 0.09 |

HAQ-DI: health assessment questionnaire disability index, CKD: chronic kidney disease, RF: rheumatoid factor, CCP: cyclic citrullinated peptide, ILD: interstitial lung disease, b/tsDMARD: biological and targeted synthetic disease modifying anti-rheumatic drug, csDMARD: conventional synthetic disease modifying anti-rheumatic drug, VAS: visualised analogue scale, EGA: evaluator’s global assessment, LDA: low disease activity, MDA: moderate disease activity, HDA: high disease activity, TNFi: tumour necrosis factor inhibitor, IL6i: interleukin-6 inhibitor, CTLA4-Ig: cytotoxic T-lymphocyte-associated antigen 4 immunoglobulin, JAKi: Janus kinase inhibitor, MTX: methotrexate, GC: glucocorticoid

**Additional file 5. Sensitivity analysis of Additional file 3 including only those whose disease duration < 5 years.**

|  | | | HAQ-DI normalization (< 0.5)  (N = 418) | | | |
| --- | --- | --- | --- | --- | --- | --- |
|  | | | OR | 95%CI | | p |
| Age | < 40 | | 1 (Reference) | | | |
|  | 40-49 | | 1.11 | 0.46 | 2.67 | 0.82 |
|  | 50-59 | | 0.40 | 0.19 | 0.86 | 0.02 |
|  | 60-69 | | 0.63 | 0.29 | 1.34 | 0.23 |
|  | 70-79 | | 0.42 | 0.19 | 0.95 | 0.04 |
|  | ≥ 80 | | 0.28 | 0.09 | 0.85 | 0.03 |
| Female | | | 0.49 | 0.29 | 0.82 | 0.01 |
| Disease duration | < 1y | | 1 (Reference) | | | |
|  | 1-2y | | 0.36 | 0.21 | 0.64 | <0.01 |
|  | 2-5y | | 0.56 | 0.33 | 0.94 | 0.03 |
| Comorbidity | Overweight | | 0.56 | 0.34 | 0.91 | 0.02 |
|  | CKD | | 1.12 | 0.58 | 2.16 | 0.74 |
|  | DM | | 1.36 | 0.85 | 2.20 | 0.20 |
|  | ILD | | 0.65 | 0.39 | 1.10 | 0.11 |
| Past history | Fracture | | 0.64 | 0.31 | 1.34 | 0.24 |
|  | Cancer | | 0.57 | 0.28 | 1.15 | 0.12 |
|  | ≥ 2 classes of b/tsDMARD failure | | 0.46 | 0.26 | 0.81 | 0.01 |
|  | ≥ 2 csDMARD failure | | 0.94 | 0.42 | 2.11 | 0.87 |
| Pre-treatment condition | HAQ-DI | | 0.21 | 0.14 | 0.31 | <0.01 |
|  | Pain VAS | | 1.00 | 0.99 | 1.02 | 0.66 |
|  | EGA | | 1.01 | 0.99 | 1.02 | 0.27 |
|  | Global Health | | 1.00 | 0.99 | 1.01 | 0.87 |
|  | Use of MTX | | 0.84 | 0.47 | 1.48 | 0.54 |
|  | Use of GC | | 1.12 | 0.63 | 2.01 | 0.70 |
|  | RF or anti-CCP positive | | 1.94 | 1.19 | 3.14 | 0.01 |
|  | Disease activity | LDA (CDAI ≤ 10) | 1 (Reference) | | | |
|  |  | MDA (10 < CDAI ≤ 22) | 0.30 | 0.11 | 0.82 | 0.02 |
|  |  | HDA (22 < CDAI) | 0.34 | 0.12 | 1.01 | 0.05 |
| Class of b/tsDMARD | TNFi | | 1 (Reference) | | | |
|  | IL6i | | 0.76 | 0.45 | 1.30 | 0.32 |
|  | ABT | | 0.75 | 0.39 | 1.41 | 0.37 |
|  | JAKi | | 2.22 | 1.05 | 4.72 | 0.04 |

b/tsDMARD: biological and targeted synthetic disease modifying anti-rheumatic drug, RF: rheumatoid factor. CCP: cyclic citrullinated peptide, CKD: chronic kidney disease, ILD: interstitial lung disease, csDMARD: conventional synthetic disease modifying anti-rheumatic drug, HAQ-DI: health assessment questionnaire disability index, VAS: visualised analogue scale, EGA: evaluator’s global assessment, CDAI: clinical disease activity index, LDA: low disease activity, MDA: middle disease activity, HDA: high disease activity, TNFi: tumour necrosis factor inhibitor, IL6i: interleukin-6 inhibitor, CTLA4-Ig: cytotoxic T-lymphocyte-associated antigen 4 immunoglobulin, JAKi: Janus kinase inhibitor, MTX: methotrexate, GC: glucocorticoid, OR: odds ratio, CI: confidence interval

**Additional file 6. Sensitivity analysis of Additional file 3 including only those who achieved CDAI remission (< 2.8).**

|  | | | HAQ normalization (< 0.5)  at one year (N = 793) | | | |
| --- | --- | --- | --- | --- | --- | --- |
|  |  |  | OR | 95%CI | | p |
| Age | < 40 | | 1 (Reference) | | | |
|  | 40-49 | | 0.64 | 0.20 | 2.02 | 0.45 |
|  | 50-59 | | 0.38 | 0.14 | 1.02 | 0.06 |
|  | 60-69 | | 0.60 | 0.22 | 1.63 | 0.32 |
|  | 70-79 | | 0.41 | 0.15 | 1.13 | 0.08 |
|  | ≥ 80 | | 0.45 | 0.11 | 1.89 | 0.28 |
| Female | | | 0.39 | 0.20 | 0.77 | 0.01 |
| Disease duration | < 1y | | 1 (Reference) | | | |
|  | 1-2y | | 0.36 | 0.15 | 0.86 | 0.02 |
|  | 2-5y | | 0.44 | 0.20 | 0.99 | 0.05 |
|  | 5-10y | | 0.68 | 0.29 | 1.61 | 0.38 |
|  | 10-20y | | 0.30 | 0.13 | 0.69 | 0.01 |
|  | > 20y | | 0.18 | 0.06 | 0.50 | <0.01 |
| Comorbidity | Overweight | | 0.81 | 0.45 | 1.47 | 0.49 |
|  | CKD | | 0.88 | 0.46 | 1.69 | 0.71 |
|  | Glucose intolerance | | 1.41 | 0.80 | 2.50 | 0.24 |
|  | ILD | | 0.62 | 0.35 | 1.11 | 0.11 |
| Past history | Fracture | | 1.72 | 0.78 | 3.80 | 0.18 |
|  | Cancer | | 0.47 | 0.23 | 0.97 | 0.04 |
|  | ≥ 2 classes of b/tsDMARD failure | | 0.48 | 0.26 | 0.90 | 0.02 |
|  | ≥ 2 csDMARD failure | | 1.10 | 0.56 | 2.14 | 0.78 |
| Pre-treatment condition | HAQ-DI | | 0.24 | 0.16 | 0.37 | <0.01 |
|  | Pain VAS | | 1.00 | 0.99 | 1.02 | 0.76 |
|  | EGA | | 1.01 | 0.99 | 1.02 | 0.47 |
|  | Global Health | | 1.01 | 0.99 | 1.02 | 0.46 |
|  | Dose of MTX | | 1.01 | 0.97 | 1.06 | 0.59 |
|  | Dose of GC | | 0.96 | 0.87 | 1.07 | 0.47 |
|  | RF or anti-CCP positive | | 2.23 | 1.21 | 4.13 | 0.01 |
|  | Disease activity | LDA (CDAI ≤ 10) | 1 (Reference) | | | |
|  |  | MDA (10 < CDAI ≤ 22) | 0.49 | 0.20 | 1.24 | 0.13 |
|  |  | HDA (22 < CDAI) | 0.51 | 0.18 | 1.47 | 0.21 |
| Class of b/tsDMARD | TNFi | | 1 (Reference) | | | |
|  | IL6i | | 0.99 | 0.50 | 1.95 | 0.98 |
|  | ABT | | 0.80 | 0.38 | 1.67 | 0.55 |
|  | JAKi | | 2.46 | 1.07 | 5.67 | 0.03 |

b/tsDMARD: biological and targeted synthetic disease modifying anti-rheumatic drug, RF: rheumatoid factor. CCP: cyclic citrullinated peptide, CKD: chronic kidney disease, ILD: interstitial lung disease, csDMARD: conventional synthetic disease modifying anti-rheumatic drug, HAQ-DI: health assessment questionnaire disability index, VAS: visualised analogue scale, EGA: evaluator’s global assessment, CDAI: clinical disease activity index, LDA: low disease activity, MDA: middle disease activity, HDA: high disease activity, TNFi: tumour necrosis factor inhibitor, IL6i: interleukin-6 inhibitor, CTLA4-Ig: cytotoxic T-lymphocyte-associated antigen 4 immunoglobulin, JAKi: Janus kinase inhibitor, MTX: methotrexate, GC: glucocorticoid, OR: odds ratio, CI: confidence interval

**Additional file 7. Background and treatment outcomes of the participants by positivity or negativity of RF/anti-CCP antibody.**

|  | | RF or anti-CCP positivity | | | | | | p* |
| --- | --- | --- | --- | --- | --- | --- | --- | --- |
|  |  | Negative  (N = 359, 19%) | | | Positive  (N = 1497, 81%) | | |  |
| **Background** | | Mean | SE | Median | Mean | SE | Median |  |
| Age | | 60.76 | 0.66 | 64 | 62.68 | 0.32 | 65 | 0.01 |
| Disease duration (month) | | 68.55 | 4.41 | 34 | 115.12 | 2.80 | 72 | <0.01 |
| BMI | | 23.39 | 0.22 | 22.49 | 22.38 | 0.09 | 21.94 | <0.01 |
| eGFR | | 75.11 | 1.17 | 74.49 | 78.06 | 0.58 | 77.84 | 0.03 |
| CDAI at week 0 | | 25.43 | 0.59 | 23.5 | 24.31 | 0.27 | 23 | 0.08 |
| SDAI at week 0 | | 27.25 | 0.66 | 25.11 | 26.25 | 0.30 | 24.365 | 0.15 |
| DAS28-ESR at week 0 | | 5.24 | 0.06 | 5.19 | 5.37 | 0.03 | 5.4 | 0.05 |
| HAQ at week 0 | | 1.14 | 0.04 | 1 | 1.22 | 0.02 | 1.125 | 0.06 |
| PainVAS at week 0 | | 51.75 | 1.17 | 52 | 51.37 | 0.60 | 52 | 0.78 |
| EGA at week 0 | | 42.17 | 0.95 | 40 | 43.34 | 0.46 | 42 | 0.26 |
| Global Health at week 0 | | 51.85 | 1.10 | 52 | 50.36 | 0.57 | 50 | 0.24 |
| Dose of MTX (mg/week) | | 8.63 | 0.28 | 10 | 8.04 | 0.15 | 8 | 0.07 |
| Dose of GC (mg/day, PSL equivalent) | | 1.32 | 0.13 | 0 | 0.99 | 0.06 | 0 | 0.01 |
| **Outcome** | |  |  |  |  |  |  |  |
| CDAI at 6 months | | 6.57 | 0.35 | 4.8 | 6.48 | 0.18 | 4.7 | 0.83 |
| SDAI at 6 months | | 6.76 | 0.36 | 5.03 | 6.80 | 0.19 | 4.86 | 0.92 |
| DAS28-ESR at 6 months | | 2.67 | 0.06 | 2.47 | 2.96 | 0.03 | 2.82 | <0.01 |
| HAQ-DI at 6 months | | 0.71 | 0.04 | 0.5 | 0.80 | 0.02 | 0.625 | 0.04 |
| Pain VAS at 6 months | | 29.39 | 1.26 | 23 | 27.66 | 0.64 | 20 | 0.23 |
| Change in clinical parameters | Δ CDAI | -19.07 | 0.67 | -17.3 | -18.15 | 0.32 | -16.8 | 0.21 |
|  | Δ SDAI | -20.76 | 0.76 | -18.635 | -19.80 | 0.35 | -18.17 | 0.24 |
|  | Δ DAS28-ESR | -2.59 | 0.08 | -2.55 | -2.43 | 0.04 | -2.425 | 0.07 |
|  | Δ HAQ-DI | -0.40 | 0.03 | -0.25 | -0.41 | 0.02 | -0.25 | 0.91 |
|  | Δ Pain VAS | -22.34 | 1.51 | -18.5 | -24.03 | 0.74 | -20 | 0.32 |
|  |  | N | % |  | N | % |  |  |
| Disease activity | Remission | 124 | 34.5 |  | 532 | 35.5 |  | 0.71 |
|  | LDA (CDAI ≤ 10) | 154 | 42.9 |  | 664 | 44.4 |  |  |
|  | MDA (10 < CDAI ≤ 22) | 69 | 19.2 |  | 249 | 16.6 |  |  |
|  | HDA (22 < CDAI) | 12 | 3.3 |  | 52 | 3.5 |  |  |
| HAQ-DI improvement | | 187 | 52.1 |  | 821 | 54.8 |  | 0.19 |
| HAQ-DI at 6 months ≤ 0.5 | | 153 | 42.6 |  | 640 | 42.8 |  | 0.97 |
| Pain VAS reduction at 6months | | 78 | 21.7 |  | 418 | 27.9 |  | 0.02 |

HAQ-DI: health assessment questionnaire disability index, BMI: body mass index, GFR: glomerular filtration rate, RF: rheumatoid factor. CCP: cyclic citrullinated peptide, CDAI: clinical disease activity index, SDAI: simplified disease activity index, DAS: disease activity score; VAS: visualised analogue scale, EGA: evaluator’s global assessment, GH: patient’s global health, MTX: methotrexate, GC: glucocorticoid, CKD: chronic kidney disease, ILD: interstitial lung disease, b/tsDMARD: biological and targeted synthetic disease modifying anti-rheumatic drug, csDMARD: conventional synthetic disease modifying anti-rheumatic drug

*Student’s t-test was used for continuous variables and chi-square test were used for categorical variables

**Additional file 8. Multiple logistic regression analysis for factors related to HAQ-DI normalization (< 0.5) at 6 months.**

|  | | | HAQ normalization (< 0.5)  (N=732) | | | |
| --- | --- | --- | --- | --- | --- | --- |
|  |  |  | OR | 95%CI | | p |
| Age | <40 | | 1 (Reference) | | | |
|  | 40-49 | | 0.59 | 0.30 | 1.16 | 0.13 |
|  | 50-59 | | 0.44 | 0.24 | 0.82 | 0.01 |
|  | 60-69 | | 0.50 | 0.27 | 0.93 | 0.03 |
|  | 70-79 | | 0.37 | 0.19 | 0.70 | <0.01 |
|  | ≥ 80 | | 0.25 | 0.10 | 0.60 | <0.01 |
| Female | | | 0.45 | 0.30 | 0.67 | <0.01 |
| Disease duration | <1y | | 1 (Reference) | | | |
|  | 1-2y | | 0.36 | 0.21 | 0.62 | 0.00 |
|  | 2-5y | | 0.50 | 0.31 | 0.83 | 0.01 |
|  | 5-10y | | 0.53 | 0.32 | 0.90 | 0.02 |
|  | 10-20y | | 0.37 | 0.22 | 0.62 | <0.01 |
|  | > 20y | | 0.21 | 0.11 | 0.40 | <0.01 |
| Comorbidities | Overweight (BMI > 25) | | 0.72 | 0.50 | 1.03 | 0.08 |
|  | CKD | | 1.04 | 0.67 | 1.61 | 0.86 |
|  | Glucose intolerance | | 1.45 | 1.01 | 2.08 | 0.04 |
|  | ILD | | 0.70 | 0.49 | 1.01 | 0.05 |
| Past history | Fracture | | 1.08 | 0.68 | 1.71 | 0.74 |
|  | Past history of cancer | | 0.81 | 0.50 | 1.33 | 0.40 |
|  | ≥ 2 classes of b/tsDMARD failure | | 0.46 | 0.32 | 0.68 | <0.01 |
|  | ≥ 2 csDMARD failure | | 1.02 | 0.67 | 1.55 | 0.93 |
| Pre-treatment condition | HAQ-DI | | 0.17 | 0.13 | 0.22 | <0.01 |
|  | Pain VAS | | 1.00 | 0.99 | 1.01 | 0.50 |
|  | EGA | | 1.01 | 1.00 | 1.02 | 0.05 |
|  | GH | | 1.00 | 0.99 | 1.01 | 0.89 |
|  | Use of MTX | | 0.99 | 0.96 | 1.02 | 0.40 |
|  | Use of GC | | 0.92 | 0.86 | 0.99 | 0.02 |
|  | RF or anti-CCP positive | | 2.10 | 1.42 | 3.12 | <0.01 |
|  | Disease activity | LDA (CDAI ≤ 10) | 1 (Reference) | | | |
|  |  | MDA (10 < CDAI ≤ 22) | 0.36 | 0.19 | 0.70 | <0.01 |
|  |  | HDA (22 < CDAI) | 0.36 | 0.17 | 0.75 | 0.01 |
| Class of b/tsDMARD | TNFi | | 1 (Reference) | | | |
|  | IL6i | | 0.93 | 0.62 | 1.41 | 0.74 |
|  | ABT | | 0.72 | 0.45 | 1.13 | 0.15 |
|  | JAKi | | 2.52 | 1.52 | 4.18 | <0.01 |

b/tsDMARD: biological and targeted synthetic disease modifying anti-rheumatic drug, RF: rheumatoid factor. CCP: cyclic citrullinated peptide, CKD: chronic kidney disease, ILD: interstitial lung disease, csDMARD: conventional synthetic disease modifying anti-rheumatic drug, HAQ-DI: health assessment questionnaire disability index, VAS: visualized analogue scale, EGA: evaluator’s global assessment, GH: patient’s global health, CDAI: clinical disease activity index, LDA: low disease activity, MDA: middle disease activity, HDA: high disease activity, TNFi: tumor necrosis factor inhibitor, IL6i: interleukin-6 inhibitor, CTLA4-Ig: cytotoxic T-lymphocyte-associated antigen 4 immunoglobulin, JAKi: Janus kinase inhibitor, MTX: methotrexate, GC: glucocorticoid, OR: odds ratio, CI: confidence interval

**Additional file 9. Proportion of the patients who achieved LDA (CDAI ≤10.0) at 6 months by age groups.**

| Age group | LDA  (CDAI < 10.0) | | MDA-HDA  (CDAI ≥ 10.0) | | Total | | p* |
| --- | --- | --- | --- | --- | --- | --- | --- |
|  | N | % | N | % | N | % |  |
| <40 | 131 | 8.9 | 23 | 6.0 | 154 | 8.3 | 0.19 |
| 40-49 | 159 | 10.8 | 49 | 12.8 | 208 | 11.2 |  |
| 50-59 | 278 | 18.9 | 86 | 22.5 | 364 | 19.6 |  |
| 60-69 | 410 | 27.8 | 96 | 25.1 | 506 | 27.3 |  |
| 70-79 | 393 | 26.7 | 98 | 25.7 | 491 | 26.5 |  |
| ≥ 80 | 103 | 7.0 | 30 | 7.9 | 133 | 7.2 |  |
| Total | 1474 | 100.0 | 382 | 100.0 | 1856 | 100.0 |  |

LDA: low disease activity, CDAI: clinical disease activity index, MDA: moderate disease activity, HDA: high disease activity

*Difference between the age groups was compared using chi-squared test.

**Additional file 10.** **Multiple logistic regression analysis for factors associated with pain VAS reduction by ≥ 40 mm at 6 months.**

|  | | | Pain VAS reduction  (N= 454, 32.1%) | | | |  |
| --- | --- | --- | --- | --- | --- | --- | --- |
|  |  |  | OR | 95%CI | | p | |
| Age | < 40 | | 1 (Reference) | | | |  |
|  | 40-49 | | 0.85 | 0.38 | 1.89 | 0.69 | |
|  | 50-59 | | 0.50 | 0.24 | 1.02 | 0.06 | |
|  | 60-69 | | 0.94 | 0.47 | 1.91 | 0.87 | |
|  | 70-79 | | 0.81 | 0.38 | 1.71 | 0.57 | |
|  | ≥ 80 | | 0.32 | 0.12 | 0.89 | 0.03 | |
| Female | | | 1.13 | 0.72 | 1.78 | 0.59 | |
| Disease duration | < 1y | | 1 (Reference) | | | |  |
|  | 1-2y | | 0.66 | 0.36 | 1.21 | 0.18 | |
|  | 2-5y | | 0.91 | 0.52 | 1.59 | 0.74 | |
|  | 5-10y | | 0.78 | 0.43 | 1.42 | 0.42 | |
|  | 10-20y | | 0.67 | 0.37 | 1.22 | 0.19 | |
|  | > 20y | | 0.84 | 0.42 | 1.68 | 0.62 | |
| Comorbidities | Overweight (BMI > 25) | | 0.84 | 0.55 | 1.29 | 0.42 | |
|  | CKD | | 1.23 | 0.75 | 2.03 | 0.41 | |
|  | Glucose intolerance | | 1.09 | 0.72 | 1.65 | 0.69 | |
|  | ILD | | 1.12 | 0.73 | 1.70 | 0.61 | |
| Past history | Fracture | | 0.44 | 0.26 | 0.75 | <0.01 | |
|  | Cancer | | 0.85 | 0.48 | 1.52 | 0.59 | |
|  | ≥ 2 classes of b/tsDMARD failure | | 0.45 | 0.29 | 0.72 | <0.01 | |
|  | ≥ 2 csDMARD failure | | 0.69 | 0.41 | 1.14 | 0.15 | |
| Pre-treatment condition | HAQ-DI | | 0.72 | 0.55 | 0.95 | 0.02 | |
|  | Pain VAS | | 1.10 | 1.09 | 1.12 | <0.01 | |
|  | EGA | | 1.00 | 0.99 | 1.02 | 0.69 | |
|  | GH | | 1.00 | 0.98 | 1.01 | 0.51 | |
|  | Use of MTX | | 0.97 | 0.94 | 1.00 | 0.07 | |
|  | Use of GC | | 0.92 | 0.85 | 0.99 | 0.03 | |
|  | RF or anti-CCP positive | | 2.00 | 1.25 | 3.22 | <0.01 | |
|  | Disease activity | LDA (CDAI ≤ 10) | 1 (Reference) | | | |  |
|  |  | MDA (10 < CDAI ≤ 22) | 1.13 | 0.33 | 3.89 | 0.84 | |
|  |  | HDA (22 < CDAI) | 1.46 | 0.40 | 5.26 | 0.57 | |
| Class of b/tsDMARD | TNFi | | 1 (Reference) | | | |  |
|  | IL6i | | 0.88 | 0.54 | 1.43 | 0.60 | |
|  | ABT | | 0.70 | 0.41 | 1.19 | 0.19 | |
|  | JAKi | | 1.80 | 1.00 | 3.24 | 0.05 | |

VAS: visualized analogue scale, b/tsDMARD: biological and targeted synthetic disease modifying anti-rheumatic drug, RF: rheumatoid factor. CCP: cyclic citrullinated peptide, CKD: chronic kidney disease, ILD: interstitial lung disease, csDMARD: conventional synthetic disease modifying anti-rheumatic drug, HAQ-DI: health assessment questionnaire disability index, VAS: visualized analogue scale, EGA: evaluator’s global assessment, GH: patient’s global health, CDAI: clinical disease activity index, LDA: low disease activity, MDA: middle disease activity, HDA: high disease activity, TNFi: tumor necrosis factor inhibitor, IL6i: interleukin-6 inhibitor, CTLA4-Ig: cytotoxic T-lymphocyte-associated antigen 4 immunoglobulin, JAKi: Janus kinase inhibitor, MTX: methotrexate, GC: glucocorticoid, OR: odds ratio, CI: confidence interval

**Additional file 11. Multiple logistic regression analysis for factors associated with pain VAS reduction by ≥40 mm** **at 1 year.**

|  | | | Pain VAS reduction by ≥ 40 mm  at 1 year (N = 404, 28%) | | | |
| --- | --- | --- | --- | --- | --- | --- |
|  |  |  | OR | 95%CI | | p |
| Age | < 40 | | 1 (Reference) | | | |
|  | 40-49 | | 0.71 | 0.31 | 1.61 | 0.41 |
|  | 50-59 | | 0.75 | 0.35 | 1.58 | 0.44 |
|  | 60-69 | | 1.31 | 0.62 | 2.74 | 0.48 |
|  | 70-79 | | 0.80 | 0.37 | 1.73 | 0.57 |
|  | ≥ 80 | | 0.63 | 0.22 | 1.86 | 0.41 |
| Female | | | 0.96 | 0.60 | 1.53 | 0.85 |
| Disease duration | < 1y | | 1 (Reference) | | | |
|  | 1-2y | | 0.63 | 0.33 | 1.18 | 0.15 |
|  | 2-5y | | 0.65 | 0.36 | 1.16 | 0.14 |
|  | 5-10y | | 0.56 | 0.30 | 1.03 | 0.06 |
|  | 10-20y | | 0.62 | 0.34 | 1.15 | 0.13 |
|  | > 20y | | 0.71 | 0.34 | 1.48 | 0.36 |
| Comorbidity | Overweight (BMI > 25) | | 0.72 | 0.46 | 1.14 | 0.16 |
|  | CKD | | 0.97 | 0.58 | 1.61 | 0.90 |
|  | Glucose intolerance | | 0.98 | 0.64 | 1.50 | 0.92 |
|  | ILD | | 1.01 | 0.66 | 1.57 | 0.95 |
| Past history | Fracture | | 0.80 | 0.46 | 1.38 | 0.43 |
|  | Cancer | | 0.97 | 0.54 | 1.74 | 0.92 |
|  | ≥ 2 classes of b/tsDMARD failure | | 0.51 | 0.32 | 0.83 | 0.01 |
|  | ≥ 2 csDMARD failure | | 0.83 | 0.49 | 1.38 | 0.47 |
| Pre-treatment condition | HAQ-DI | | 0.81 | 0.61 | 1.08 | 0.15 |
|  | Pain VAS | | 1.10 | 1.08 | 1.12 | <0.01 |
|  | EGA | | 1.00 | 0.98 | 1.01 | 0.64 |
|  | Global Health | | 1.00 | 0.98 | 1.01 | 0.55 |
|  | RF or anti-CCP positive | | 1.25 | 0.76 | 2.03 | 0.38 |
|  | Dose of MTX | | 0.99 | 0.96 | 1.02 | 0.46 |
|  | Dose of GC | | 0.93 | 0.85 | 1.01 | 0.07 |
|  | Disease activity | LDA (CDAI ≤ 10) | 1 (Reference) | | | |
|  |  | MDA (10 < CDAI ≤ 22) | 0.91 | 0.30 | 2.76 | 0.86 |
|  |  | HDA (22 < CDAI) | 1.15 | 0.35 | 3.73 | 0.82 |
| Class of b/tsDMARD | TNFi | | 1 (Reference) | | | |
|  | IL6i | | 0.75 | 0.45 | 1.23 | 0.26 |
|  | ABT | | 0.74 | 0.43 | 1.28 | 0.28 |
|  | JAKi | | 1.31 | 0.71 | 2.43 | 0.38 |

VAS: visualised analogue scale, HAQ-DI: health assessment questionnaire disability index, CKD: chronic kidney disease, RF: rheumatoid factor, CCP: cyclic citrullinated peptide, ILD: interstitial lung disease, b/tsDMARD: biological and targeted synthetic disease modifying anti-rheumatic drug, csDMARD: conventional synthetic disease modifying anti-rheumatic drug, EGA: evaluator’s global assessment, LDA: low disease activity, MDA: moderate disease activity, HDA: high disease activity, TNFi: tumour necrosis factor inhibitor, IL6i: interleukin-6 inhibitor, CTLA4-Ig: cytotoxic T-lymphocyte-associated antigen 4 immunoglobulin, JAKi: Janus kinase inhibitor, MTX: methotrexate, GC: glucocorticoid

**Additional file 12. Sensitivity analysis of Additional file10 including only those with disease duration < 5 years.**

|  | | | Pain VAS reduction by ≥ 40mm | | | |
| --- | --- | --- | --- | --- | --- | --- |
|  |  |  | (N = 256, 35%) | | | |
|  |  |  | OR | 95%CI | | p |
| Age | < 40 | | 1 (Reference) | | | |
|  | 40-49 | | 1.42 | 0.40 | 5.01 | 0.59 |
|  | 50-59 | | 0.52 | 0.19 | 1.48 | 0.22 |
|  | 60-69 | | 1.02 | 0.35 | 3.01 | 0.97 |
|  | 70-79 | | 0.91 | 0.29 | 2.86 | 0.88 |
|  | ≥ 80 | | 0.60 | 0.16 | 2.28 | 0.45 |
| Female | | | 1.49 | 0.82 | 2.71 | 0.19 |
| Disease duration | < 1y | | 1 (Reference) | | | |
|  | 1-2y | | 0.58 | 0.30 | 1.10 | 0.09 |
|  | 2-5y | | 1.02 | 0.53 | 1.96 | 0.96 |
| Comorbidity | Overweight | | 0.57 | 0.33 | 1.00 | 0.05 |
|  | CKD | | 0.65 | 0.30 | 1.40 | 0.27 |
|  | DM | | 1.00 | 0.56 | 1.77 | 0.99 |
|  | ILD | | 1.11 | 0.58 | 2.09 | 0.75 |
| Past history | Fracture | | 0.45 | 0.21 | 0.94 | 0.04 |
|  | Cancer | | 0.69 | 0.31 | 1.53 | 0.36 |
|  | ≥2 classes of b/tsDMARD failure | | 0.49 | 0.26 | 0.95 | 0.04 |
|  | ≥2 csDMARD failure | | 0.74 | 0.28 | 1.94 | 0.54 |
| Pre-treatment condition | HAQ-DI | | 0.79 | 0.52 | 1.20 | 0.27 |
|  | Pain VAS | | 0.98 | 0.96 | 0.99 | 0.01 |
|  | EGA | | 1.01 | 0.99 | 1.03 | 0.36 |
|  | Global Health | | 0.99 | 0.98 | 1.01 | 0.49 |
|  | Use of MTX | | 1.20 | 0.62 | 2.33 | 0.59 |
|  | Use of GC | | 1.43 | 0.70 | 2.95 | 0.33 |
|  | RF or anti-CCP positive | | 1.80 | 1.03 | 3.17 | 0.04 |
|  | Disease activity | LDA (CDAI ≤ 10) | 1 (Reference) | | | |
|  |  | MDA (10 < CDAI ≤ 22) | 0.23 | 0.03 | 1.90 | 0.17 |
|  |  | HDA (22 < CDAI) | 0.30 | 0.03 | 2.56 | 0.27 |
| Class of b/tsDMARD | TNFi | | 1 (Reference) | | | |
|  | IL6i | | 1.29 | 0.65 | 2.57 | 0.47 |
|  | ABT | | 1.11 | 0.49 | 2.54 | 0.80 |
|  | JAKi | | 0.76 | 0.33 | 1.75 | 0.52 |

b/tsDMARD: biological and targeted synthetic disease modifying anti-rheumatic drug, RF: rheumatoid factor. CCP: cyclic citrullinated peptide, CKD: chronic kidney disease, ILD: interstitial lung disease, csDMARD: conventional synthetic disease modifying anti-rheumatic drug, HAQ-DI: health assessment questionnaire disability index, VAS: visualised analogue scale, EGA: evaluator’s global assessment, CDAI: clinical disease activity index, LDA: low disease activity, MDA: middle disease activity, HDA: high disease activity, TNFi: tumour necrosis factor inhibitor, IL6i: interleukin-6 inhibitor, CTLA4-Ig: cytotoxic T-lymphocyte-associated antigen 4 immunoglobulin, JAKi: Janus kinase inhibitor, MTX: methotrexate, GC: glucocorticoid, OR: odds ratio, CI: confidence interval

**Additional file 13. Sensitivity analysis of Additional file 10 including only those who achieved CDAI remission (< 2.8).**

|  | | | Pain VAS reduction by ≥ 40 mm (N = 272, 43.7%) | | | |
| --- | --- | --- | --- | --- | --- | --- |
|  |  |  | OR | 95%CI | | p |
| Age | <40 | | 1 (Reference) | | | |
|  | 40-49 | | 0.67 | 0.12 | 3.86 | 0.66 |
|  | 50-59 | | 0.22 | 0.04 | 1.14 | 0.07 |
|  | 60-69 | | 0.69 | 0.15 | 3.17 | 0.63 |
|  | 70-79 | | 0.36 | 0.07 | 1.89 | 0.23 |
|  | ≥ 80 | | 0.16 | 0.01 | 2.35 | 0.18 |
| Female | | | 1.42 | 0.50 | 4.07 | 0.51 |
| Disease duration | <1y | | 1 (Reference) | | | |
|  | 1-2y | | 0.51 | 0.12 | 2.08 | 0.35 |
|  | 2-5y | | 0.41 | 0.12 | 1.42 | 0.16 |
|  | 5-10y | | 1.02 | 0.23 | 4.54 | 0.98 |
|  | 10-20y | | 0.59 | 0.16 | 2.20 | 0.43 |
|  | > 20y | | 0.27 | 0.04 | 1.73 | 0.17 |
| Comorbidity | Overweight (BMI > 25) | | 1.21 | 0.42 | 3.53 | 0.72 |
|  | CKD | | 1.38 | 0.45 | 4.23 | 0.57 |
|  | Glucose intolerance | | 0.77 | 0.26 | 2.32 | 0.65 |
|  | ILD | | 1.40 | 0.49 | 4.05 | 0.53 |
| Past history | Fracture | | 1.01 | 0.23 | 4.34 | 0.99 |
|  | Cancer | | 0.94 | 0.24 | 3.70 | 0.93 |
|  | ≥ 2 classes of b/tsDMARD failure | | 0.63 | 0.19 | 2.06 | 0.44 |
|  | ≥ 2 csDMARD failure | | 0.68 | 0.21 | 2.19 | 0.52 |
| Pre-treatment condition | HAQ-DI | | 0.97 | 0.47 | 1.98 | 0.92 |
|  | Pain VAS | | 1.21 | 1.15 | 1.26 | <0.01 |
|  | EGA | | 0.99 | 0.95 | 1.02 | 0.41 |
|  | Global Health | | 1.02 | 0.99 | 1.05 | 0.31 |
|  | Dose of MTX | | 0.89 | 0.82 | 0.97 | 0.01 |
|  | Dose of GC | | 1.00 | 0.79 | 1.26 | 0.98 |
|  | RF or anti-CCP positive | | 3.13 | 1.04 | 9.46 | 0.04 |
|  | Disease activity | LDA (CDAI ≤ 10) | 1 (Reference) | | | |
|  |  | MDA (10 < CDAI ≤ 22) | 0.52 | 0.08 | 3.37 | 0.49 |
|  |  | HDA (22 < CDAI) | 0.95 | 0.12 | 7.53 | 0.96 |
| Class of b/tsDMARD | TNFi | | 1 (Reference) | | | |
|  | IL6i | | 0.77 | 0.23 | 2.56 | 0.67 |
|  | ABT | | 0.46 | 0.14 | 1.53 | 0.21 |
|  | JAKi | | 1.84 | 0.47 | 7.21 | 0.38 |

factor. CCP: cyclic citrullinated peptide, CKD: chronic kidney disease, ILD: interstitial lung disease, csDMARD: conventional synthetic disease modifying anti-rheumatic drug, HAQ-DI: health assessment questionnaire disability index, VAS: visualised analogue scale, EGA: evaluator’s global assessment, CDAI: clinical disease activity index, LDA: low disease activity, MDA: middle disease activity, HDA: high disease activity, TNFi: tumour necrosis factor inhibitor, IL6i: interleukin-6 inhibitor, CTLA4-Ig: cytotoxic T-lymphocyte-associated antigen 4 immunoglobulin, JAKi: Janus kinase inhibitor, MTX: methotrexate, GC: glucocorticoid, OR: odds ratio, CI: confidence interval

**Additional file 15. Variance inflation factors (VIF) for each variable in the multiple regression test for HAQ-DI improvement.**

|  |  | | VIF | 1/VIF |
| --- | --- | --- | --- | --- |
| Age | < 40 | | Reference | |
|  | 40-49 | | 2.07 | 0.48 |
|  | 50-59 | | 2.7 | 0.37 |
|  | 60-69 | | 3.39 | 0.29 |
|  | 70-79 | | 3.61 | 0.28 |
|  | ≥ 80 | | 2.05 | 0.49 |
| Female | | | 1.09 | 0.92 |
| Disease duration | < 1y | | Reference | |
|  | 1-2y | | 1.46 | 0.69 |
|  | 2-5y | | 1.73 | 0.58 |
|  | 5-10y | | 1.82 | 0.55 |
|  | 10-20y | | 1.99 | 0.50 |
|  | > 20y | | 1.68 | 0.60 |
| Comorbidity | Overweight (BMI > 25) | | 1.04 | 0.96 |
|  | CKD | | 1.32 | 0.76 |
|  | DM | | 1.17 | 0.85 |
|  | ILD | | 1.16 | 0.87 |
| Past history | Fracture | | 1.14 | 0.88 |
|  | Cancer | | 1.09 | 0.92 |
|  | ≥ 2 classes of b/tsDMARD failure | | 1.44 | 0.69 |
|  | ≥ 2 csDMARD failure | | 1.27 | 0.79 |
| Pre-treatment condition | HAQ | | 1.63 | 0.61 |
|  | Pain VAS | | 3.09 | 0.32 |
|  | EGA | | 2.06 | 0.49 |
|  | Global Health | | 2.91 | 0.34 |
|  | Use of MTX | | 1.41 | 0.71 |
|  | Use of GC | | 1.12 | 0.89 |
|  | RF or anti-CCP positive | | 1.06 | 0.94 |
|  | Disease activity | LDA (CDAI ≤ 10) | Reference | |
|  |  | MDA (10 < CDAI ≤ 22) | 4.01 | 0.25 |
|  |  | HDA (22 < CDAI) | 5.48 | 0.18 |
| Class of b/tsDMARD | TNFi | | Reference | |
|  | IL6i | | 1.49 | 0.67 |
|  | ABT | | 1.45 | 0.69 |
|  | JAKi | | 1.52 | 0.66 |
| **Mean VIF : 1.95** | | | | |

CKD: chronic kidney disease, RF: rheumatoid factor, CCP: cyclic citrullinated peptide, ILD: interstitial lung disease, b/tsDMARD: biological and targeted synthetic disease modifying anti-rheumatic drug, csDMARD: conventional synthetic disease modifying anti-rheumatic drug, HAQ-DI: health assessment questionnaire disability index, CDAI: clinical disease activity index, VAS: visualised analogue scale, EGA: evaluator’s global assessment, LDA: low disease activity, MDA: moderate disease activity, HDA: high disease activity, TNFi: tumour necrosis factor inhibitor, IL6i: interleukin-6 inhibitor, CTLA4-Ig: cytotoxic T-lymphocyte-associated antigen 4 immunoglobulin, JAKi: Janus kinase inhibitor, MTX: methotrexate, GC: glucocorticoid
